# Alterations in fecal *Lactobacillus* and *Bifidobacterium* species in type 2 diabetic patients in Southern China population

**DOI:** 10.3389/fphys.2012.00496

**Published:** 2013-01-31

**Authors:** Kim-Anne Lê, Yan Li, Xiaojing Xu, Wanting Yang, Tingting Liu, Xiaoning Zhao, Yongming Gorge Tang, Dehong Cai, Vay Liang W. Go, Stephen Pandol, Hongxiang Hui

**Affiliations:** ^1^International Center for Metabolic Diseases, Southern Medical UniversityGuangzhou, China; ^2^Nutrition and Health Department, Nestec Ltd., Nestlé Research CenterLausanne, Switzerland; ^3^Dongguan SMU Metabolic Medicine Inc. Ltd.Dongguang, China; ^4^School Biotechnology, Southern Medical UniversityGuangzhou, China; ^5^Department of Endocrinology, Zhujiang Hospital Southern Medical UniversityGuangzhou, China; ^6^Center of Metabolic Diseases, Beijiao Hospital, Southern Medical UniversityGuangzhou, China; ^7^Department of Medicine, Cedars-Sinai Medical CenterLos Angeles, CA, USA; ^8^Department of Medicine, VA Greater Los Angeles Health Care SystemLos Angeles, CA, USA; ^9^UCLA Center for Excellence in Pancreatic Diseases, David Geffen School of Medicine at University of California, Los AngelesLos Angeles, CA, USA

**Keywords:** microbiota, microflora, metabolic diseases, gut

## Abstract

**Background:** The connection between gut microbiota and metabolism and its role in the pathogenesis of diabetes are increasingly recognized. The objective of this study was to quantitatively measure *Bifidobacterium* and *Lactobacillus* species, members of commensal bacteria found in human gut, in type 2 diabetic patients (T2D) patients from Southern China.

**Methods:** Fifty patients with T2D and thirty control individuals of similar body mass index (BMI) were recruited from Southern China. T2D and control subjects were confirmed with both oral glucose tolerance test (OGTT) and HbA_1c_ measurements. *Bifidobacterium* and *Lactobacillus* species in feces were measured by real-time quantitative PCR. Data were analyzed with STATA 11.0 statistical software.

**Results:** In comparison to control subjects T2D patients had significantly more total *Lactobacillus* (+18%), *L. bugaricus* (+13%), *L. rhamnosum* (+37%) and *L. acidophilus* (+48%) (*P* < 0.05). In contrast, T2D patients had less amounts of total *Bifidobacteria* (−7%) and *B. adolescentis* (−12%) (*P* < 0.05). Cluster analysis showed that gut microbiota pattern of T2D patients is characterized by greater numbers of *L. rhamnosus* and *L. acidophillus*, together with lesser numbers of *B. adolescentis* (*P* < 0.05).

**Conclusion:** The gut microflora in T2D patients is characterized by greater numbers of *Lactobacillus* and lesser numbers of *Bifidobacterium* species.

## Introduction

The pathophysiology of type 2 diabetes (T2D) involves both environmental and genetic factors. Recently, the gut microflora has emerged as another parameter at the crossroad of these interactions. Several animal and human studies have demonstrated that the gut microflora composition differs between T2D and controls, and may play a role in the development of insulin resistance and T2Ds (Cani et al., 2008; Delzenne and Cani, 2011). In addition, it has been shown that modification of the gut microbiota by environmental factors may alter body weight and energy metabolism regulation, which may lead to the development of obesity, the major risk factor for T2D (Backhed et al., 2004; Tremaroli et al., 2010).

More than 10^12^ microorganisms can be found in the human colon (Eckburg et al., 2005; Andersson et al., 2008). Of these, the *Firmicutes, Bacteroides, Actinobacteria*, and *Proteobacteria phyla* are the most prominent (Arumugam et al., 2011). These bacteria can be categorized as commensal, beneficial, or harmful. Some of the deleterious effects include activation of inflammatory processes, alteration of the intestinal barrier, and modification of metabolic pathways (Cani et al., 2008); while beneficial effects include anti-inflammatory effects, improved nutrient digestion, and absorption and regulation of lipid metabolism (Russell et al., 2011). Until now, most studies investigating the gut microbiota composition have used large throughput screening methods to distinguish specific patterns between various patient types (Larsen et al., 2010). One report indicates that *Bifidobacterium* is underrepresented in T2D patients compared to controls, and may possibly play a role in the development of T2D (Wu et al., 2010b).

*Bifidobacterium* and *Lactobacillus* belong to the *Actinobacteria* and *Firmicutes phyla*, respectively. In addition to the fact that these genera are highly prevalent in the human gut, they also are added as probiotics in foods, making them therefore ideal candidates for potential clinical interventions. Effects of the various strains of bacteria on health can be very divergent, and it still remains unclear which species are responsible for specific metabolic effects. In mice, administration of *Lactobacillus casei* improves diet-induced obesity and insulin resistance (Naito et al., 2011). However, it has also been shown that presence of certain *Lactobacillus* species may increase inflammation, which may be related to obesity and T2D (Zeuthen et al., 2006; Santacruz et al., 2009). Increasing the content of gut *Bifidobacterium* resulting from a prebiotic dietary fiber intervention improved high-fat diet-induced glucose intolerance, insulin secretion, and low-grade inflammation (Cani et al., 2007). Until now, most studies have been performed in Caucasian populations leaving unknown whether the gut microflora may have similar relationships to metabolism in other ethnicities.

Therefore, the primary aim of this study was to determine if there are specific species differences in total *Lactobacillus, L. acidophilus, L. bulgaricus, L. casei, L. rhamnosus, L. plantarum, Total Bifidobacterium, B. longum, B. breve, B. adolescentis, and B. infantis* between T2D and control patients in Southern China.

## Materials and methods

### Study subjects

Diabetic subjects and control subjects were selected for this prospective study based on inclusion criteria listed below between March 2008 and January 2010 at Southern Medical University in Guangzhou, Southern China. Subjects with normal oral glucose tolerance test (OGTT) and HbA1C measurements (see below) were selected as controls. Fifty T2D patients and thirty control subjects of broad age (20–76 years) and body mass index (BMI) (17–36 kg/m^2^) ranges were included in this study. Patients were defined as T2D if their fasting and 2-h OGTT glucose values were ≥7.0 mmol/l and ≥11.1 mmol/l respectively, and if they had disease duration of at least 5 years duration but otherwise healthy, age 18–80 years, male or female, stable glycemic control (mean HbA_1c_ levels <8.0%).

All T2D patients had made lifestyle modifications and 21/50 patients took medications, such as metformin, acarbose, glyburide or Chinese herbal medicine (Pueraria skullcap Coptis soup), and achieved glycemic control (Hb_A1c_ of 6–7%, Preprandial blood glucose: 4.0–6.0 mmol/L, 2-h postprandial blood glucose: 5.0–8.0 mmol/L.), While the control subjects with normal OGTT had A1C of 4.2–6.3% (mean, 5.4%). All patients and control subjects were selected from Southern Medical University hospitals; and most of them lived in the same community and ate a southern Chinese style diet twice a day.

Participants were excluded if there was any evidence of diarrhea, constipation, significant cardiovascular complications, microalbuminuria (>40 mg per 24 h) or frank proteinuria, evidence of unstable glycemic control (mean HbA_1c_ levels > 8.0%), or history of admission for hyperglycemia or hypoglycemia in the 6 months prior to recruitment, other significant renal, hepatic, cardiovascular, or neurological disease; cancer; pregnancy. All participants provided their signed written consent and this study protocol was approved by the Ethical Committee of Southern Medical University.

### Anthropometry and metabolic quantifications

Weight and height were measured to the nearest 0.1 kg and 0.1 cm, respectively, using a beam medical scale and wall-mounted stadiometer; and BMI was calculated as previously described (Zeuthen et al., 2006). All patients stopped taking their diabetes medications for 3 days and had overnight fast before investigation Glucose tolerance was evaluated using 75 g oral dose of glucose. Venous blood samples were drawn twice from antecubital vein. The first sample was drawn at 8–10 AM after overnight fasting for at least 12 h, and the second samples was drawn 2 h after 75 g oral glucose loading. The first blood sample was examined for baseline metabolic profiles [hemoglobin (HbA1c, C-peptide, C-reactive protein, fasting blood glucose, cholesterol, triglyceride, high-density lipoprotein cholesterol (HDL-C), low-density lipoprotein cholesterol (LDL-C))]. The second blood sample was examined for blood glucose and insulin levels. Fecal specimens were collected within 12 h after the administration of oral glucose for subsequent measurement of bacterial composition.

#### Blood analysis

Blood samples were centrifuged immediately for 10 min at 2500 RPM at 8–10°C. Then the serum was removed and frozen at −70°C until assayed. Glucose was assayed in duplicate on a Yellow Springs Instrument 2700 Analyzer (Yellow Springs Instrument, Yellow Springs, OH) using the glucose oxidase method, and Glycated hemoglobin (HbA1c) was measured by using a HPLC cation exchange column method (Modular Diabetic Monitoring System; Bio-Rad, Richmond, CA). C-peptide was determined by ELISA (DRG-Diagnostica, Marburg, Germany).

#### Extraction of bacterial DNA from fecal samples

Total bacterial DNA was extracted from the fecal samples using DNA stool kit according to the manufacture's protocol (*Qiagen*, Valencia, CA, USA). DNA concentration and quality in the extracts was determined by agarose gel electrophoresis. Altogether, nine species of probiotic bacteria were chosen for measurement including *L. acidophilus, L. bulgaricus, L. casei, L. rhamnosus, B. breve, B. longum*, and *B. infantis*. Specific forward and reverse primers for each bacterium were designed (Table 1).

**Table 1:** **PCR primers for detection of *Bifidobacterium* and *Lactobacillus***.

**Gut flora**	**Forward primer 5′–3′**	**Reverse primer 5′–3′**	**Size (bp)**	**References**
*Lactobacillus*	AGCAGTAGGGAATCTTCCA	CACCGCTACACATGGAG	341	Armougom et al., 2009
*Lactobacillus acidophilus*	GAAAGAGCCCAAACCAAGTGATT	CTTCCCAGATAATTCAACTATCGCTTA	145	Wellen and Hotamisligil, 2005
*Lactobacillus bulgaricus*	GGRTGATTTGTTGGACGCTAG	GCCGCCTTTCAAACTTGAATC	138	Wu et al., 2010a
*Lactobacillus casei*	GCACCGAGATTCAACATGG	GGTTCTTGGATYTATGCGGTATTAG	122	Wu et al., 2010a
*Lactobacillus rhamnosus*	TGCTTGCATCTTGATTTAATTTTG	GTCCATTGTGGAAGATTCCC	317	Wu et al., 2010a
*Lactobacillus plantarum*	TGGATCACCTCCTTTCTAAGGAAT	TGTTCTCGGTTTCATTATGAAAAAATA	145	Haarman and Knol, 2006
*Bifidobacterium*	GCGTGCTTAACACATGCAAGTC	CACCCGTTTCCAGGAGCTATT	126	Armougom et al., 2009
*Bifidobacterium longum*	GAGACAGAAACTTTCGAAGC	GAAGTCTGTGGTATCCAATCC	112	Byun et al., 2004
*Bifidobacterium breve*	TTCCGCATTCGTGTTATTGA	CACATCTTCGCTATCCAGCA	279	Byun et al., 2004
*Bifidobacterium adolescentis*	CTCCAGTTGGATGCATGTC	CGAAGGCTTGCTCCCAGT	122	Matsuki et al., 1998
*Bifidobacterium infantis*	CCATCTCTGGGATCGTCGG	TATCGGGGAGCAAGCGTGA	563	Matsuki et al., 1998

#### Real-time qPCR

Bacterial copy numbers in fecal samples from 50 subjects with T2D and 30 controls were quantified by qPCR using the 7500 Fast Real-time PCR system (Applied Biosystems, USA). The qPCR reaction mixture (20 μl) was composed of each 0.6 μl forward and reverse primer, 6.4 μl sterile ddH_2_O, ROX Reference Dye (50 X) 0.4 μl, SYBR premix DimerEraser (2 X) 10 μl (Takara, Biotechnology, Dalian, China) and 2 μl fecal DNA as template added in 10-fold serial dilutions from 10^2^ to 10^12^ copy/ml. The amplification program consisted of one cycle of 95°C for 30s, followed by 40 cycles of 95°C for 5 s, 55°C for 30 s, and 72°C for 34 s. Each fecal sample measurement was performed in duplicate. Standard curves were constructed using 10-fold serial dilutions of fecal bacterial DNA of known concentration. Copy numbers of bacteria in fecal samples were calculated from the threshold cycle values (Ct) and expressed as quantity of bacteria per gram feces. Since data were not normally distributed, these values were subsequently transformed using logarithm.

### Statistical methods

All data are means ± SD. Statistical analyses were performed using STATA 11.0 (Stata Corp, College Station, TX). *P*-values <0.05 was considered statistically significant. Values for bacterial content were transformed using logarithm in order to reach a normal distribution. Unadjusted paired comparisons were done using Student *t*-tests. Adjustments of comparisons for age and gender were performed using analysis of covariance (ANCOVA). Bacterial content patterns were evaluated using cluster analysis of observations, which separates participants into mutually exclusive groups and maximizes differences in the content of a number of bacteria. The cluster k-means procedure in STATA, version 11.0, was used, based on the K-means method. The relationship between the two clusters identified and the presence of diabetes was tested using the chi-squared test. Differences among clusters were investigated using the Kruskal–Wallis analysis of variance test.

## Results

A total of 30 control participants (43% females) and 50 patients with T2D (54% females) completed the study. Anthropometric characteristics of the two groups are presented in Table 2. T2D patients were significantly older than the control group (60 ± 8 vs. 41 ± 11 years, *P* < 0.001), and subsequent analyses were thus adjusted for age. T2D patients had significantly greater amount of the total *Lactobacillus*, as well as greater amounts of *L. bulgaricus*, *L. casei, L. rhamnosus* and *L. acidophillus*, compared to controls (Table 3). However, when adjusted for age and gender, the differences for *L. casei* were no longer significant. T2D patients had less numbers of total *Bifidobacterium* and *B. adolescentis* than controls. These differences remained significant when adjusted for age and gender.

**Table 2:** **Anthropometric parameters**.

**Parameter**	**Control (*n* = 30)**	**Diabetic (*n* = 50)**	***P*-value**
Sex (M/F)	17/13	23/27	0.3
Age (years)	41 ± 11	60 ± 8	<0.001
Weight (kg)	64.2 ± 9.0	62.9 ± 10.0	0.7
BMI (kg/m^2^)	23.9 ± 3.0	24.7 ± 3.8	0.5

**Table 3:** **Comparisons of bacteria amounts between controls and diabetic patients (bacterial values transformed using logarithm)**.

	**Control (*n* = 30)**	**Diabetic (*n* = 50)**	**Unadjusted *P*-value**	***P*-value adjusted for age and gender**
***Lactobacillus***				
*Lactobacillus bulgaricus*	4.6 ± 0.8	5.2 ± 0.6	0.0006	0.03
*Lactobacillus casei*	4.5 ± 0.9	5.0 ± 1.0	0.03	0.19
*Lactobacillus plantarum*	4.6 ± 0.7	4.7 ± 0.9	0.9	0.68
*Lactobacillus rhamnosus*	2.7 ± 0.4	3.7 ± 1.0	<0.0001	0.004
*Lactobacillus acidophillus*	2.9 ± 0.6	4.3 ± 1.4	<0.0001	0.01
*Lactobacillus* (total)	6.6 ± 0.5	7.8 ± 0.9	<0.0001	0.0005
***Bifidobacterium***				
*Bifidobacterium longum*	3.9 ± 0.8	3.7 ± 0.7	0.15	0.2
*Bifidobacterium breve*	3.9 ± 0.3	3.9 ± 0.3	0.4	0.4
*Bifidobacterium adolescentis*	4.0 ± 0.3	3.5 ± 0.4	<0.0001	0.0003
*Bifidobacterium infantis*	3.9 ± 1.0	4.0 ± 0.9	0.7	0.3
*Bifidobacterium* (total)	11.6 ± 0.9	10.7 ± 0.7	<0.0001	0.002

**Figure 1:**
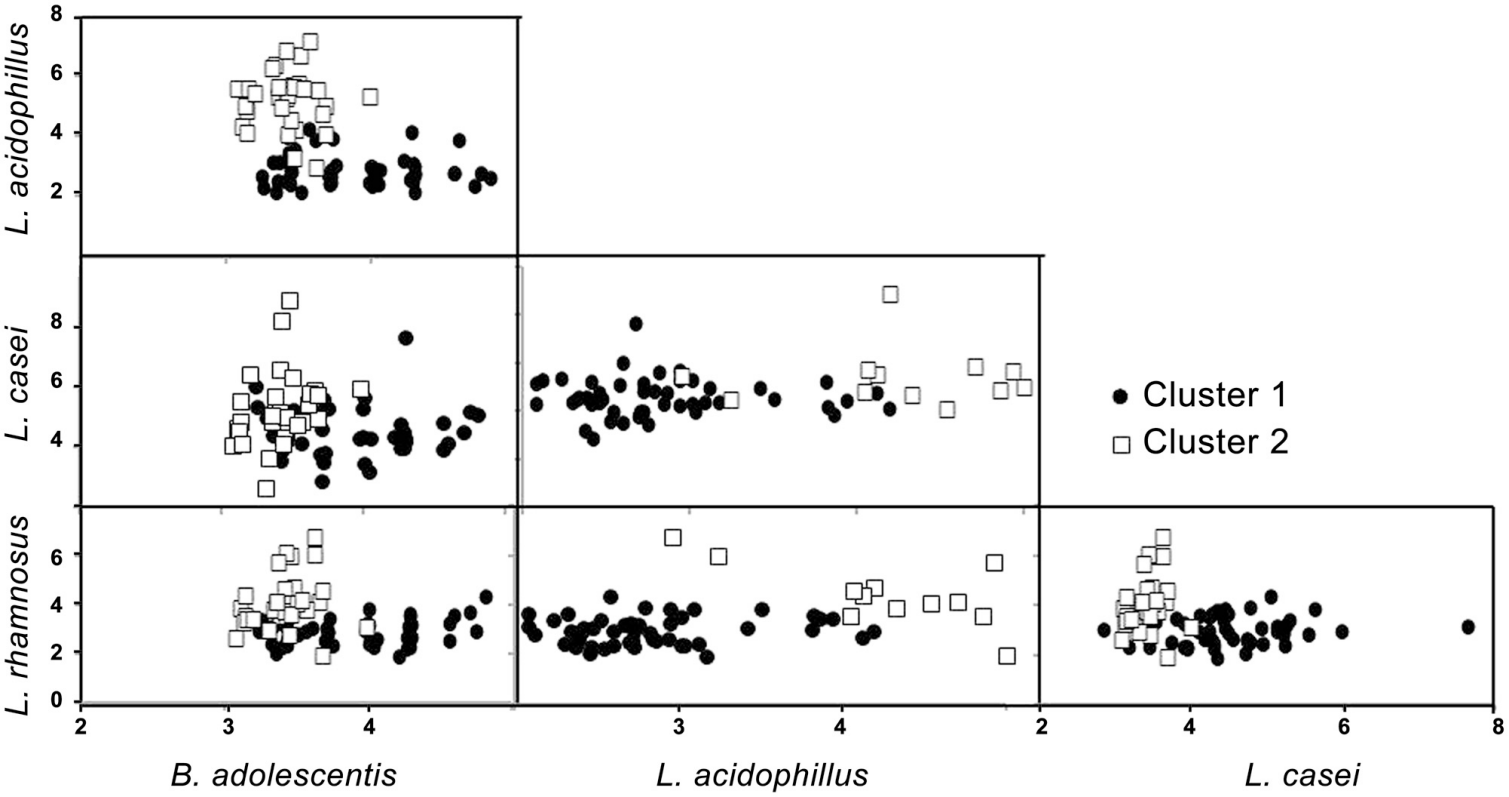
**Schematic representation of the two clusters (bacterial values transformed using logarithm)**.

Cluster analysis identified two mutually exclusive clusters characterized as follows: cluster 1 had greater numbers of *L. rhamnosus* and *L. acidophillus*, and a lesser numbers of *B. adolescentis* (Figure 1 and Table 4). Cluster 2 had the opposite description, i.e., lesser numbers of *L. rhamnosus* and *L. acidophillus*, and a greater numbers of *B. adolescentis*. Interestingly, there was a strong association between the clusters and the presence of diabetes (*p*-value of chi-squared test: *P* < 0.001), suggesting that the presence of diabetes may be characterized by a specific gut microflora pattern. Thus, greater numbers of *L. rhamnosus* and *L. acidophillus*, and a lesser numbers of *B. adolescentis*, are high specificity descriptors of Cluster 1 for T2D. Sensitivity and specificity calculations of this cluster analysis showed that for such bacterial analysis and prediction of T2D, the sensitivity = 0.62, specificity = 0.97, positive predictive value = 0.97 and negative predictive value = 0.60.

**Table 4:** **Comparisons of bacteria amount by clusters (bacterial values transformed using logarithm)**.

	**Cluster 1 (*n* = 32)**	**Cluster 2 (*n* = 48)**	**Unadjusted *P*-value**	***P*-value adjusted for age and gender**
Cases of Diabetes (%)	31 (97%)	19 (40)	<0.0001	
***Lactobacillus***				
*Lactobacillus bulgaricus*	5.1 ± 0.6	4.9 ± 0.8	0.09	0.01
*Lactobacillus casei*	5.2 ± 1.2	4.5 ± 0.8	0.003	0.2
*Lactobacillus plantarum*	4.8 ± 0.9	4.6 ± 0.8	0.21	0.6
*Lactobacillus rhamnosus*	4.0 ± 1.0	2.9 ± 0.5	<0.0001	0.0001
*Lactobacillus acidophillus*	5.1 ± 0.9	2.8 ± 0.5	<0.0001	<0.0001
*Lactobacillus* (total)	7.7 ± 0.9	7.0 ± 0.9	0.001	0.02
***Bifidobacterium***				
*Bifidobacterium longum*	3.5 ± 0.7	3.9 ± 0.8	0.06	0.6
*Bifidobacterium breve*	3.9 ± 0.3	3.9 ± 0.3	0.4	0.2
*Bifidobacterium adolescentis*	3.4 ± 0.2	3.9 ± 0.4	<0.0001	0.003
*Bifidobacterium infantis*	3.9 ± 0.8	4.0 ± 1.0	0.5	0.7
*Bifidobacterium* (total)	10.7 ± 0.7	11.2 ± 0.9	0.03	0.4

Patients took multiple medications including metformin, the prescriptions were personalized so that there were different doses of medications in variety combinations. We compared metformin group with other patients group, no difference has been observed.

## Discussion

Previous studies have shown that specific gut microbiota composition is linked to the presence of T2D (Larsen et al., 2010; Wu et al., 2010b). In the present study, we showed that in a Southern Chinese population, T2D patients have increased numbers of *L. Bulgaricum*, *L. rhamnosus and L. acidophillus* and decreased numbers of *B. Adolescentis*, compared to controls. Together, this set of bacteria formed a cluster characteristic of T2D patients.

Previous studies have shown that T2D patients had greater numbers of *Lactobacillus* and lesser numbers of *Bifidobacterium* in their gut as measured in feces compared to non-diabetic patients. Our results obtained in Chinese subjects are in line with these studies. In addition, we further measured individual species of *Lactobacillus* and *Bifidobaterium* and showed that several *Lactobacillus* species, namely *L. bulgaricum, L. rhamnosus and L. acidophillus* are increased in number in T2D patients. The role of *Lactobacillus* in metabolic diseases remains unclear. Administration of *L. casei* strain improved glucose tolerance in diet-induced obese mice (Naito et al., 2011). In contrast, other studies point to a pro-inflammatory role of *Lactobacillus*. For example, obese individuals have greater numbers of *Lactobacillus* in their feces, compared to lean controls (Armougom et al., 2009). Obesity is characterized by a chronic, low-grade inflammation, which may lead to the development of insulin resistance and ectopic fat deposition (Wellen and Hotamisligil, 2005). Although the mechanisms remain unknown, it has been speculated that modifications of the gut microbiota may increase local and plasma lipopolysaccharide (LPS), which may in turn lead to inflammation, hepatic fat accumulation, insulin resistance, and T2D (Cani et al., 2008). In our study, *Lactobacillus* levels were not related to plasma C-reactive proteins concentrations, a measure of inflammation (data not shown). However, presence of such bacteria may locally induce inflammation or other cytokines that we did not measure, such as TNF-α or IL-6.

We also showed that *Bifidobacterium* numbers were decreased in T2D patients. Our results support previous studies, showing that both insulin resistant and obese patients have decreased *Bifidobacterium* numbers in their gut (Larsen et al., 2010; Wu et al., 2010b). In the present study, the only species that was significantly different was *B. adolescentis. B. adolescentis* has been shown to reduce intestinal permeability, thus providing a better protection against endotoxin translocation (Wu et al., 2010a). Previous studies suggest that high-fat diet-induced endotoxemia could modify gut microbiota, and lead to a decrease in *Bifidobacterium* species (Cani et al., 2007). The pathogenesis of T2D is strongly influenced by environmental factors, such as diet. Therefore, it remains possible that poor nutritional habits may in the long term contribute to modification of the gut microbiota, which may in turn play a role in the development of T2D. Further mechanistic studies will be required to assess causal relationships.

Based on these various differences in gut microbiota composition between T2D patients and the control individuals, we further aimed to assess whether T2D could be characterized by a specific “bacterial signature.” Using cluster analysis, we identified two mutually exclusive clusters. Cluster 1 was characterized by high amount of *L. Rhamnosus* and *L. acidophillus*, while lower amount of *B. adolescentis*. Interestingly, all except one individual in Cluster 1 had T2D, suggesting that such analysis can provide high specificity for the disease. Indeed, the specificity for such test was 0.97, and the positive predictive value reached the same score of 0.97. This suggests that most individuals identified with Cluster 1 have a high probability of being diabetic. However, the number of cases of “false negative” remained high, with a sensitivity of 0.62 and a negative predictive value of 0.60. Of note, it is not clear why one individual in Cluster 1 did not have diabetes. However, considering the very high sensitivity of Cluster in patients with diabetes, it is conceivable that this one patient represents the metabolic phenotype of diabetes without the usual clinical measures of diabetes. On the other hand, the high false negative rate of Cluster 1 designation for diabetes patients suggests that Cluster 1 designation represents a unique metabolic subset of patients with diabetes. These suggestions may lead to further investigations to understand the mechanisms of these differences in the patient groups. Therefore, more accurate evaluation and refined analysis of gut bacteria will need to be performed. In addition, the relationship between the changes of the gut microbiota and the development of T2Ds should be investigated in a prospective cohort study, which would be useful to identify individuals at high risk for T2D. Although these preliminary results need to be further validated by larger number of patients and more refined bacterial analysis, they may provide some direction for development of future diagnostic tool to identify individuals at high risk for T2D and initiate early prevention and treatment.

The potential clinical impacts of this study are 2-fold: first, we have identified a bacterial signature of T2D, which may be further developed and possibly used as a future diagnostic tool for identification of patients at risk for T2D. Second, our findings may lead to dietary recommendations for gut microbiota modulation such as diets promoting increased *Bifidobacterium*. In conclusion, we showed that T2D patients can be characterized by a specific fecal bacterial pattern of *Lactobacillus* and *Bifidobacterium species*. Modification of these bacteria levels, may influence T2D.

### Conflict of interest statement

Kim-Anne Lê is employed by Nestec Ltd., which is a subsidiary of Nestlé Ltd. and provides professional assistance, research, and consulting services for food, dietary, dietetic, and pharmaceutical products of interest to Nestlé Ltd. The other authors declare that the research was conducted in the absence of any commercial or financial relationships that could be construed as a potential conflict of interest.
